# Repeat Element Activation-Driven Inflammation: Role of NFκB and Implications in Normal Development and Cancer?

**DOI:** 10.3390/biomedicines10123101

**Published:** 2022-12-01

**Authors:** Baptiste Dumetier, Camille Sauter, Azadeh Hajmirza, Baptiste Pernon, Romain Aucagne, Cyril Fournier, Céline Row, Fabien Guidez, Cédric Rossi, Côme Lepage, Laurent Delva, Mary B. Callanan

**Affiliations:** 1 Faculty of Medicine, INSERM1231, University of Burgundy, 21000 Dijon, France; 2 Institute for Research in Immunology and Cancer, Montreal, QC H3C 3J7, Canada; 3 Unit for Innovation in Genetics and Epigenetics in Oncology, Dijon University Hospital, 21000 Dijon, France; 4 CRIGEN, Crispr-Functional Genomics, Dijon University Hospital and University of Burgundy, 21000 Dijon, France; 5 School of Medicine, Stanford University, Stanford, CA 94305, USA

**Keywords:** repetitive elements, histone H3 lysine 9 trimethylation, satellite repeats, viral mimicry, innate immunity, immune checkpoint blockade, RNA-guided chromatin regulation, p53

## Abstract

The human genome is composed of unique DNA sequences that encode proteins and unique sequence noncoding RNAs that are essential for normal development and cellular differentiation. The human genome also contains over 50% of genome sequences that are repeat in nature (tandem and interspersed repeats) that are now known to contribute dynamically to genetic diversity in populations, to be transcriptionally active under certain physiological conditions, and to be aberrantly active in disease states including cancer, where consequences are pleiotropic with impact on cancer cell phenotypes and on the tumor immune microenvironment. Repeat element-derived RNAs play unique roles in exogenous and endogenous cell signaling under normal and disease conditions. A key component of repeat element-derived transcript-dependent signaling occurs via triggering of innate immune receptor signaling that then feeds forward to inflammatory responses through interferon and NFκB signaling. It has recently been shown that cancer cells display abnormal transcriptional activity of repeat elements and that this is linked to either aggressive disease and treatment failure or to improved prognosis/treatment response, depending on cell context and the amplitude of the so-called ‘viral mimicry’ response that is engaged. ‘Viral mimicry’ refers to a cellular state of active antiviral response triggered by endogenous nucleic acids often derived from aberrantly transcribed endogenous retrotransposons and other repeat elements. In this paper, the literature regarding transcriptional activation of repeat elements and engagement of inflammatory signaling in normal (focusing on hematopoiesis) and cancer is reviewed with an emphasis on the role of innate immune receptor signaling, in particular by dsRNA receptors of the RIG-1 like receptor family and interferons/NFκB. How repeat element-derived RNA reprograms cell identity through RNA-guided chromatin state modulation is also discussed.

## 1. Introduction

Epigenetic plasticity and dysregulated cell fate decisions are increasingly recognized as major drivers of malignant transformation, clonal evolution and treatment resistance in cancer [1]. Epigenetic information is relayed by chemical modifications to DNA, RNA (methylation, for example) and histones (acetylation, methylation, for example) and via the regulated spatial organization of chromatin domains in the nucleus, notably heterochromatin and euchromatin. Maladaptive epigenetic responses, linked to genetic (mutations in genes encoding epigenetic regulators or histone genes, for example) and nongenetic mechanisms, are functionally implicated in the emergence of the premalignant state and to clonal evolution and treatment resistance thereafter. Indeed, epigenetic dysregulation is known to drive cancer cell fate/metabolic switches that override environmental and oncogenic stress responses, tumor suppressor mechanisms, and immune surveillance systems [1,2]. Thus, identification and therapeutic intervention of epigenetic malfunction in cancer is an attractive option for personalized patient treatment, particularly in immuno-oncology [2]. Among the more recent mechanisms to gain attention in the field of cancer epigenetics are those that are triggered by aberrant repeat sequence transcriptional activation. This awakening of repeat sequence transcription in cancer cells can shape cancer cell phenotypes through chromatin-dependent mechanisms and by induction of antiviral-type signaling responses in a process now referred to as ‘viral mimicry’ [3]. The latter occurs through detection of endogenous cytosolic self-nucleic acids (RNA or DNA)—in this instance RE-derived—by innate pattern recognition receptors (PRRs) and consequent constitutive activation of inflammatory responses, which are known to impact responses to immune-based anticancer therapies, a process that has remained underappreciated until recently [3]. Similarly, induction of this viral mimicry response in cancer cells may facilitate responses to immune-based anticancer therapies such as immune checkpoint blockade [3].

Establishment of heterochromatin that is chemically ‘marked’ by histone H3 lysine 9 trimethylation (H3K9me3) is a major pathway in the suppression of unscheduled repeat element activation and is a significant barrier to cellular reprogramming and genome instability [4]. Indeed, H3K9me3 chromatin domains assemble into nuclear foci (frequently referred to as ‘condensates’) through a process termed liquid–liquid phase separation (LLPS), These nuclear domains are intimately involved in gene and repeat element silencing [5] and are thus an important facet of the organization of the nuclear microenvironment [6]. Silencing of RE activity is also critically dependent on DNA methylation and posttranscriptional mechanisms, such as RNA editing or RNA methylation that target RE-derived RNA stability and function [3].

Transcriptional activation of RE, particularly pericentric repeats, is a recognized component of the cell stress response [7]. RE activation and expression of RE-derived RNA is gathering recognition as a crucial component for control of chromatin dynamics during normal development and cellular differentiation [8,9,10,11] and in disease states such as cancer [3]. Indeed, ectopic activation of repeat element transcription is an established characteristic of many cancers where it seems to impact on many of the canonical cancer hallmarks from cell fate decision-making, genome instability, inflammation, and tumor immune surveillance by innate and adaptive immune responses (see below). Outcomes appear to be dependent on the specific repertoire of repeat element RNAs that are expressed (“repeatome”) in a given cancer cell context and to what degree compensatory epigenetic control and ‘viral mimicry’ responses are engaged [3]. It should also be noted that evasion of the viral mimicry response is an acquired characteristic of certain cancers, such as acute myeloid leukemia (via SETDB1) [12] and treatment-resistant triple-negative breast cancer (via EZH2) [13], thus evoking new therapeutic vulnerabilities in these malignancies.

Mechanisms involved in either scheduled or unscheduled de-silencing of repeat elements are not fully understood, but—at least in cancer—disordered H3Kme3 signaling to chromatin is implicated [3], as is aberrant DNA methylation [5,14]. It is also of note that p53 and BRCA tumor suppressor proteins function in repeat element silencing [15,16], as does pRB/EZH2 [17] and the transcription factor PLZF [18].

More detailed investigations of RE silencing/de-silencing mechanisms in normal and disease states are warranted in view of the key role of RE in homeostatic control of tissue function and the fact that repeat element activation in cancer cells induces chromatin reprogramming and chronic inflammatory signaling via interferon and NFκB which together are responsible for tissue microenvironment remodeling (immune and stromal cells) downstream of the viral mimicry response [3].

Exploration of RE activation status in normal cellular systems and in cancer is thus emerging as a powerful means for discovery of new cell signaling and disease mechanisms of interest for development of novel biomarkers and treatment strategies for precision oncology.

Following an overview of RE organization in the genome and RE activity, this review examines the functional role of repeat element transcripts (repeatome) in diverse systems from development, cellular senescence, hematopoiesis and cancer, with special emphasis on RE-derived RNA-mediated inflammatory signaling and RNA-guided chromatin regulation. Recent literature regarding the repeatome and its functional and biomarker value will be discussed in the context of cancer with special emphasis on the interplay between heterochromatin malfunction, aberrant cell fate decisions, viral mimicry, and clinical correlates.

## 2. Overview of Repeat Elements: Genomic Organization and Transcriptional Activity

### 2.1. Genomic Organization of Repeat Elements

**Figure 1:**
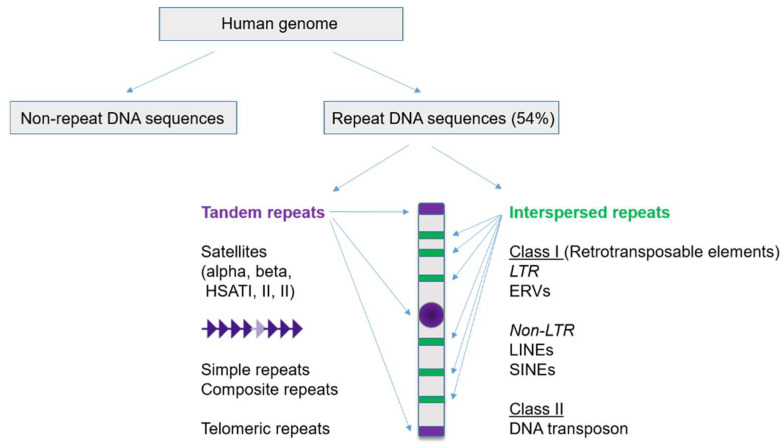
Schematic representation of the classification of repetitive elements in the human genome. These comprise more than 50% of the human genome and are divided into tandem and interspersed repeats, respectively. Among the tandem repeats are those that are enriched at centromeres, pericentromeres and telomeres (schematized as purple chromatin domains on the chromosome cartoon). Interspersed repeats—indicated as the green chromatin domains on the chromosome cartoon (not to scale)—include the endogenous retroelements and DNA transposons. The interspersed repeats are present along chromosome arms, including near gene promoters, where they may contribute to gene regulation, or are localized in intergenic regions. The interspersed repeats are further divided into class I and II repeats, as indicated. Abbreviations: SINE, short interspersed element; ERV, endogenous retrovirus; LTR, long terminal repeat; SVA, SINE—variable number tandem repeat—Alu; LINE, long interspersed element (see text for further details).

Repeat elements and repetitive DNA comprise about 50% of the human genome and are classified into two main types on the basis of their genomic organization: tandem repeats and interspersed repeats (Figure 1). Tandem repeats are enriched and structurally important for maintenance of centromere, pericentromere, and telomere stability. The tandem repeats are further subdivided into satellites and simple repeats with satellites often further classed by their regional chromosomal distribution (centromeric, pericentric, for example). Centromeric (ALR for alpha-like repeats, and GSAT for gamma satellites) and pericentric satellite repeats (BSR for beta satellite repeats, and HSATI, II and HSATIII) together make up around 6.2% of the human genome, according to the recent Telomere-to-Telomere Consortium study [19]. The classical human satellite DNAs, HSAT, also referred to as human satellites 1, 2 and 3 (HSat1, HSat2, HSat3, or collectively HSat1–3), occur on most human chromosomes as large, pericentromeric tandem repeat arrays, (100 megabases, on average) [20]. Even though HSat1–3 were among the first human DNA sequences to be isolated and characterized, they have remained almost entirely missing from the human genome reference assembly for 20 years, thus hindering studies of their sequence, regulation, and potential structural roles in the nucleus. Recently, the Telomere-to-Telomere Consortium produced the first truly complete assembly of a human genome [21], paving the way for new studies of HSat1–3 with modern genomics [20] and their impact on human health and disease.

Interspersed repeats largely refer to transposable elements (TEs) that are classified on the basis of their mechanism of propagation. Class I elements are spread within genomes via RNA-dependent retrotransposition (RTE, for retrotransposable elements) and are further subdivided into two broad subclasses: long interspersed nuclear elements (LINEs, also called LINE-1 or L1, accounting for about 22% of repeats) and long terminal repeat (LTR) elements (or ERVs for endogenous retrovirus elements), which (when active) typically encode their own catalyzing enzymes (Figure 1). The other subclass of RTE consists of short interspersed elements (SINEs) (13% approximately of repeats) and the composite retroelement SINE-VNTR (variable number of tandem repeats)-Alus (SVAs), both of which are nonautonomous, relying on LINE1-encoded proteins for retrotransposition (Figure 1). Class II TEs are those that are mobilized through DNA intermediates via transposase, helicase, or recombinase (Figure 1). It should be stressed that many repeat sequences are derived from ancient retroviruses that have integrated into the human genome throughout evolution [21]. Although most repeats are silent in somatic tissue, it is estimated that between 10% and 15% are actively transcribed in diverse physiological and disease settings [22]. Among the retrotransposons, most are inactive for retrotransposition due to accumulation of mutations along the coding region and epigenetic silencing; however, some remain active and can be a source of genome instability, particularly in cancer [23].

### 2.2. Repeat Element Activity in Chromatin Organization, Gene Regulation and Innate Immune Signaling: State of the Art

During evolutionary time, host cell functions have evolved mechanisms to suppress deleterious activity of RE, in particular retrotransposons, while co-opting benefits from RE, in a process referred to as exaptation [24]. Disturbances in this evolutionary balance are associated with aging and cancer [24]. As an example, regardless of transposition competence, RTE-derived RNA has been shown to be transcribed in early development [25,26], cell differentiation [9,11], stress [7], aging and autoimmunity [24], and cancer [3,27,28], where it is shown to play a role in shaping chromatin organization, regulation of protein-coding genes, and in triggering of innate immune-receptor signaling, depending on the cellular context.

Regarding RE activity in chromatin organization and regulation of gene activity, it is now known that RE can contribute to DNA regulatory sequences, such as transcription factor binding sites, that directly regulate neighboring genes and gene networks in innate immunity [29] and in embryogenesis [26]. Likewise, genomic RE sequences themselves or RE-derived transcripts can contribute to transcriptional control of nearby genes, either in cis or in trans, via gene recruitment into defined nuclear neighborhoods [24,26], and this process appears to be co-opted in cancer cells to regulate gene expression programs and cancer cell phenotypes, such as epithelial-mesenchymal transition [30], the drug-tolerant state [31] or aberrant gene silencing by position effect variegation-like processes (gene silencing by proximity to heterochromatin) and/or altered chromatin organization [32,33].

**Figure 2:**
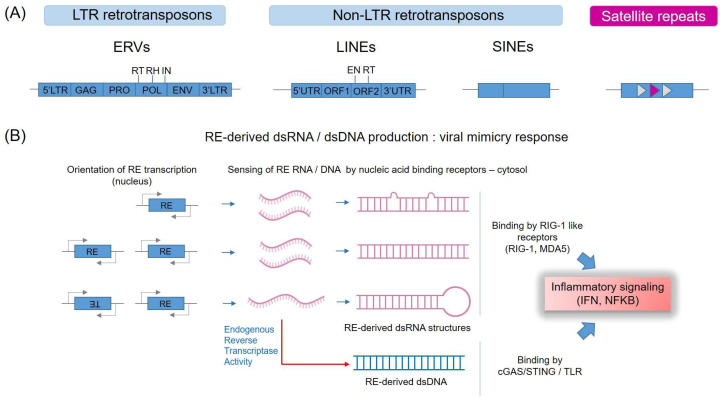
Viral mimicry initiation. (**A**) Endogenous retrotransposon element (RTE) classes and satellite repeats. Transcripts derived from families of endogenous retroviral elements (ERV) (LTR retrotransposons), non-LTR retrotransposons (LINEs, SINEs which are polyadenylated, not shown) and satellite repeats have been implicated in the viral mimicry response. The RTE encode overlapping Gag (capsid protein), Pro (protease), Pol (polymerase), and Env (envelope) ORFs (open reading frames), although many human ERVs lack envelope proteins and multiple germline mutations have inactivated ORFs. In contrast, some human LINEs and SINE families are mobile due to L1 ORF1p and ORF2p activity that targets both young LINE and SINE RNAs for retrotransposition (readers can see a review for further details [24]). Amongst other features, Pol incorporates reverse transcriptase (RT), ribonuclease H (RH), and integrase (IN) activities, as depicted. (**B**). Endogenous retroelement (ERE) transcripts can form dsRNAs. dsRNAs can form in trans through bidirectional transcription of a single RTE locus or through RNAs transcribed from the same or similar elements from different loci, where dsRNAs can form through imperfect base pairing, as indicated. dsRNAs can also form in cis through transcription of inverted-repeat (IR) elements with a single transcript, and subsequent base pairing to form a stem loop dsRNA structure. Endogenous reverse transcriptase activity can convert target RT or other RE RNA to dsDNA. These nucleic acids can trigger the viral mimicry response by PRR (pathogen pattern recognition receptors) leading to NFκB and interferon inflammatory signaling; dsRNA products are recognized by RIG-1-like receptors, as indicated. By contrast, dsDNA products are recognized and trigger signaling by the cGAS/STING/TLR PRR, as indicated (figure adapted from, [3]).

Regarding immune signaling, it has become evident that once exported to the cytoplasm, RE-derived RNA (or indeed DNA, produced consequently to endogenous retrotranscriptase activity) can directly trigger innate immune receptor signaling and inflammatory responses through the presence in RE sequences of pathogen-associated features that are more typically found in immunostimulatory viral RNA and DNA [34,35] (Figure 2).

As discussed in detail below, members of the RIG-1 like family of dsRNA binding receptors are major mediators of this self-RNA driven viral mimicry response [36]. Likewise, RE RNA that has been retrotranscribed by endogenous reverse transcription activity into double-stranded DNA (dsDNA) in the cytosol can trigger signaling by dsDNA-binding innate immune receptor pathways, such as the cyclic GMP-AMP synthase (cGAS) stimulator of IFN genes (STING) pathway [37,38]. The immunostimulatory and proinflammatory properties of RE-derived RNA are gaining increasing interest as therapeutic vulnerabilities in cancer [3].

**Figure 3:**
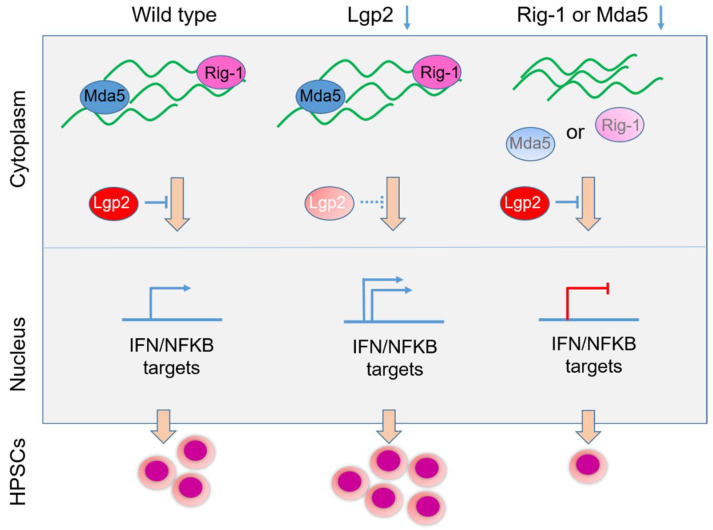
Proposed mechanism for repeat element RNA-dependent activation of the innate immune receptors Rig-1 and Mda5 and negative control by Lgp2 in hematopoietic stem and progenitor cells. Inflammatory signaling is a key regulator of developmental hematopoiesis, but the mechanisms driving such signals have remained unknown. Recent studies in mouse and zebrafish systems have provided insight into the underlying mechanisms by revealing endogenous repetitive element RNAs as activators of the pathogen pattern recognition receptors (PRR) of the RIG-I-like receptor family during embryonic hematopoiesis. RIG-I-like receptor activation induces inflammatory signals necessary for hematopoietic stem and progenitor cell generation (**left** panel). Lgp2 deficiency (LGP2 encoded by the *DHX58* gene in humans) enhances inflammatory signaling via IFN/NFκB and HSPC formation (**center** panel). Rig-I or Mda5 deficiency impairs inflammatory signaling and HSPC formation (**right** panel). Overexpression of repeat element RNA (specifically a SINE RNA that is schematically represented in green) enhances HSPC formation by engaging Rig-I and Mda5 (adapted from [9] and described in detail below).

In the following sections, the impact of RE activation on chromatin function and the viral mimicry response (acute or chronic) are discussed in more detail, in the context of physiological systems, from development, hematopoiesis (Figure 3), cellular senescence as well as in selected cancer models.

## 3. Repeat Element Activation in Normal Development, Senescence and Cellular Differentiation: Emerging Paradigms

Transcriptional activity of repeat elements, particularly HSATII/III, has long been known to be induced under cell stress conditions such as heat shock. The heat shock factor 1 transcription factor HSF1 is known to control this process, which—together with induction of HSPs (heat shock proteins)—is believed to represent a means for stressed cells to rapidly sequester RNA polymerase II away from protein-coding genes, thereby allowing cellular stress to resolve [7]. This process has been reviewed in detail elsewhere and will not be further discussed here.

### 3.1. RE-Derived RNA in Development: Role in Chromatin Organization and Control of Gene Expression

In normal development, expression of RE-derived RNAs (satellites and retrotransposons) has been described in early embryogenesis. For example, a burst of pericentric repeat expression has been shown to permit assembly of structures called chromocenters, which are masses of heterochromatin—densely packed DNA and proteins—that come together during the interphase of the cell cycle in very early development, and this is required for developmental progression, at least in mice [25]. In keeping with these findings, the Torres–Padilla laboratory has shown that de novo heterochromatin establishment during preimplantation mammalian development (starting in the male pronucleus, immediately after fertilization) is regulated by pericentromeric RNA and its ability to negatively regulate the activity of the histone methyl transferase Suvar39h2, which together allows establishment of initially non-repressive H3K9me3 [39]. Forced early deposition of H3K9me3 leads to abnormal developmental and epigenetic reprogramming [39]. Later in mammalian development, RE-derived RNA is implicated in the definition of chromatin states that ‘mark’ or directly orchestrate regulation of developmental stage-specific protein-coding genes with specific functions [26]. Indeed, SINE, L1, and low-complexity repeats have been shown to be nonrandomly distributed across the genome and to ‘barcode’ genes according to functional category thereby contributing to their temporal regulation. Remarkably, L1-enriched genes are sequestered into inactive nucleolus or lamina-associated domains and silenced in an L1-RNA-dependent manner. L1 RNA thus controls the nuclear localization and repression of L1-enriched genes during development [26].

### 3.2. Repeat Element Transcript-Activated Innate Immune Receptor Signaling, NFκB, Inflammation and Hematopoiesis

Hematopoietic cells, and especially the lymphoid cell lineages, are among the best-characterized models of lineage-dependent differentiation [40,41,42]. In addition to the activity of the well-defined transcription factors in the establishment and maintenance of the hematopoietic and progenitor stem cell compartment in development and its somatic maintenance thereafter, it is now emerging that sterile inflammatory signaling via innate immune receptor signaling activated by repeat-derived transcripts and NFκB play a key role in stress-induced hematopoiesis in various settings. Specifically, transposable elements are emerging as potent responders of stress stimuli that affect the self-renewal capacity of hematopoietic stem cells [9,10,11].

#### 3.2.1. Inflammation and Hematopoiesis

Hematopoietic stem and progenitor cells (HSPCs) are formed in vertebrates through an endothelial-to-hematopoietic transition. Following the primitive hematopoietic wave that produces the first erythrocytes and myeloid cells, HSPCs emerge during the definitive wave in the aorta–gonad–mesonephros region [43,44]. Basal inflammatory signaling has been shown in several studies to regulate HSPC development during embryogenesis [45,46,47]. For instance, signaling downstream of the cytokines tumor necrosis factor a (TNF-a) and interferon (IFN), as well as Toll-like receptor 4 (TLR4), affect HSPC emergence in both mouse and zebrafish [48,49,50,51]. More recently, the function of RIG-I-like receptors (RLRs), in HSPC development has been explored, and this has led to the discovery of a novel cytoplasmic activation mechanism for these receptors via repeat element transcript binding by these receptors (Figure 3).

#### 3.2.2. RIG1 Receptors and Hematopoiesis

The following sections provide an overview of the functional role of RIG1 receptors and their role in repeat transcript-activated RIG receptor signaling to NFκB/IFN in hematopoietic development and in hematopoietic rescue after chemotherapy.

##### RIG-1-like Receptors

The RIG-I-like receptors (RLRs) are a family of three innate immune receptors, namely, retinoic acid inducible gene I (RIG-I), melanoma differentiation-associated protein 5 (MDA5), and laboratory of genetics and physiology 2 (LGP2) (encoded by the DHX58 gene), capable of recognizing viral RNA and initiating antiviral responses. Activation of these RIG-I-like receptors (RLRs) results in their binding to MAVS, a transmembrane protein on the surface of mitochondria that serves as an adapter between the sensors and the kinase TBK1, and downstream effectors such as the IRF3/7 transcription factors, or regulators of NFκB, thus activating type I IFN production and proinflammatory cytokine production [36].

All three members of the RLR family are RNA helicases, but RIG-I and MDA5 also contain two caspase activation and recruitment domains (CARDs) that are needed for signal transduction. LGP2, by contrast, does not engage a downstream activation pathway because of its CARD-free structure and has thus been described as a positive or negative regulator of the RLR signaling pathway [36].

RLRs can also be activated also by self-elements including self-RNAs in some instances. Indeed, mitochondrial RNA [52] and endogenous repetitive element-derived transcripts can activate RLRs. Importantly, cytosolic retroelement-derived dsRNA has been shown to activate RLRs in hematopoietic development where inflammatory signaling by interferons and NFκB is induced [9,11], and in various solid cancers [3,53,54] where it can shape the cancer immune microenvironment and thus responses to immune checkpoint inhibitor therapy, for example (discussed further below).

##### RIG Receptors and Hematopoiesis: Role of Repeat Element Transcripts and Interferon and NFκB Signaling

Here we discuss in detail some major recent findings showing that IFN and NFκB-dependent inflammatory signaling, triggered by RE-derived dsRNA activation of RIG receptor signaling, is required for hematopoietic stem and progenitor cell (HSPC) normal development and HSPC recovery from chemotherapy [9,11].

By formulating the hypothesis that self-RNA derived from TE might trigger RIG1 receptor activation, Trompouki and colleagues investigated the involvement of RIG-I-like receptors (RLRs) in HSPC formation [9]. By using a zebrafish model, the investigators showed that Rig-I or Mda5 deficiency impaired and Lgp2 deficiency (negative regulator of Rig-I and Mda5) enhanced HSPC emergence in zebrafish embryos. This was related to inflammatory signaling that was reduced in Rig-I- or Mda5-deficient conditions and enhanced in Lgp2-deficient embryos [9]. Simultaneous reduction of Lgp2 and either Rig-I or Mda5 rescued inflammatory signals and HSPC numbers [9] (Figure 3).

More detailed investigations by expression profiling and assay for transposase-accessible chromatin using sequencing (ATAC-seq) revealed multiple thousands of open chromatin domains that were selectively ‘opened’ upon Rig-1 or Mda5 activation by RE transcripts [9,26]. These chromatin domains fell mostly within intergenic, intronic, and transcription start site-proximal regions, which by de novo and known motif analysis were found to harbor potential binding sites for hematopoietic (GATA, RUNX motifs) and inflammatory (IRF, P65/RELA motifs) transcription factors [9,26]. Further analysis revealed that 48% of deregulated genes for rig-I- and 46% for mda5-deficient embryos, exhibited changes (reduced) in chromatin accessibility. Conversely, lgp2-deficient embryos had increased chromatin accessibility in regions harboring the hematopoietic and inflammatory factor motifs IRF and P65/RELA, indicative of a negative regulatory role by lgp2 over Rig-1 and Mda5 inflammatory signaling in hematopoiesis [9,26]. This was supported by further assays in an NFκB-GFP reporter zebrafish model, which showed lgp2 regulatory function to operate through attenuation of inflammatory signaling. A striking finding was that repetitive element transcripts could be detected in hemogenic endothelial cells and HSPCs (nucleus and cytoplasm), suggesting a dual role as RLR ligands with possible nuclear functions as well. In keeping with the former, ectopic expression of repetitive elements enhanced HSPC formation in wild type, but not in Rig-I- or Mda5-deficient embryos [9,26] (Figure 3).

Importantly, manipulation of RLR expression in mouse fetal liver HSPCs indicated functional conservation of this novel pathway among species. Thus, repetitive elements transcribed during development drive RLR-mediated inflammatory signals mediated by NFκB/P65 and IFN that regulate HSPC formation [9,26].

Taken together, during the endothelial-to-hematopoietic transition, where cells transition from one cell type to the next, different repetitive elements are expressed and trigger inflammatory signaling. Trompouki and colleagues have speculated that while this transition is happening, newly expressed repetitive elements are sensed by RLRs and thus actively participate in shaping developmental fate. This process would be akin to the so-called hypertranscription [8]. Other nucleic acid sensors, such as the DNA sensor STING or the RNA sensor TLR3, could potentially also function in this system. In sum, this landmark study indicates a role for dsRNA repeat element-derived transcription and inflammatory signaling in the control of blood cell development.

##### Transposable Element Transcript-Driven Activation of MDA5 for Enhanced Hematopoietic Regeneration after Chemotherapy

The hematopoietic system, though tightly regulated, is vulnerable to both intrinsic and extrinsic factors that influence HSPC fate. Although HSPCs are normally quiescent, mechanisms have evolved to respond to cell stress [11]. One such mechanism that has been recently described concerns the role of increased chromatin reorganization and transcription of TE elements during early hematopoietic recovery after chemotherapy [11]. In the latter study, TE transcripts were found to bind to and activate the innate immune receptor MDA5 to generate an inflammatory response that is necessary for HSCs to exit quiescence. Indeed, HSCs that lack MDA5 were found to show an impaired inflammatory response after chemotherapy and to maintain their quiescence, with consequent better long-term repopulation capacity. Remarkably, overexpression of ERV and LINE superfamily TE copies in wild-type HSCs, but not in Mda5-/- HSCs, resulted in their cycling while knockdown of LINE1 family copies in HSCs resulted in maintenance of quiescence [11]. Thus, TE transcripts act as ligands that activate MDA5 during hematopoietic regeneration, thereby enabling HSCs to mount an inflammatory response necessary for their exit from quiescence.

### 3.3. RE Activity in Senescence-Associated Inflammatory Signaling

As discussed above, RNA derived from endogenous retrotransposable elements can also be retrotranscribed to form dsDNA by active LINE-1 element-encoded retrotranscriptase (product of the ORF2 segment of LINE1 elements, see Figure 2). The resultant dsDNA is also immunostimulatory and can induce inflammatory responses. For example, during cellular senescence, LINE1-derived transcripts have been shown to be induced and retrotranscribed by the LINE-1-encoded reverse transcriptase, leading to expression of cytoplasmic LINE1 complementary DNA (cDNA) [55]. The latter can trigger an interferon response via the cytosolic dsDNA-binding receptors cGAS and Sting [55]. Importantly, this response can be antagonized by nucleoside reverse transcriptase inhibitors (NRTIs) that inhibit the LINE-1 reverse transcriptase. Interestingly, the investigators found that treatment of aged mice with the NRTI lamivudine downregulated IFN-I activation and age-associated inflammation in several tissue types. Thus, retrotransposon activation is an important component of sterile inflammation (also called ‘inflammaging’), a hallmark of aging. LINE-1 retrotransposon activity could be a relevant target for the treatment of age-associated disorders [55]. Similar mechanisms operate in oncogene-induced senescence [55], are aberrantly activated, at least in colon cancer [37], as discussed below, and may be responsible for the autoimmune signaling in diseases such as systemic lupus, where LINE-1 elements have been shown to be transcriptionally active [56] or in Setdb1-deficient models of inflammatory bowel disease, where ERV elements show derepression [57].

## 4. Repeat Element Activation in Cancer: Functional Impact on Cancer Cell Phenotypes and Immune Signaling

Repeat element activation has been shown to be a feature of cancer cells [27,28,34,58]. An emerging paradigm is that the cancer cell ‘repeatome’ shapes cellular phenotypes, functional states and response to anticancer therapy, in particular sensitivity to immune checkpoint blockade through engagement of viral mimicry responses. Indeed, a recurring feature upon activation of repeat elements in cancer cells is engagement of inflammatory signaling (via NFκB/IFN), alongside ‘oncogene driver’ signatures suggestive of a role for ectopic repeat element transcription in the conditioning of both immune contexture and cell fate decisions (aberrant) in cancer cell populations and tissue. While mechanisms of derepression of repeat elements in cancer remain to be explored in detail, a role for H3K9me3 malfunction and defective control by key epigenetic regulators, such as the histone methyl transferase SETDB1 [59], DNA methylation [53,54,60], and members of the PRMT family of arginine methyltransferase, are formally implicated in a number of settings [31] as is TP53 [61,62], BRCA1 [16], Rb/EZH2 [17] and PLZF [18], as mentioned earlier.

### 4.1. Overview of Viral Mimicry in Solid Cancers and Acute Myeloid Leukemia

In the following sections, selected papers that describe the cell intrinsic and extrinsic impact of RE activation in cancer are discussed in detail.

#### 4.1.1. Pancreatic Ductal Adenocarcinoma (PDAC)

In an elegant study using flow cytometry-purified epithelial cells from human PDAC and normal pancreas, respectively, the genome-wide transcriptome and DNA methylome landscapes were derived for this disease. By clustering analysis based on DNA methylation, two distinct PDAC groups displaying different methylation patterns at regions encoding repeat elements were discovered; methylation ‘low’ PDAC (named methylation cluster 1; MC1) and methylation ‘high’ tumors (named methylation cluster 2; MC2) [63]. Methylation^low^ tumors were found to be characterized by higher expression of endogenous retroviral transcripts, specifically LTR/ERV and LINE, but not SINE (satellite sequence expression was not examined), as well as double-stranded RNA sensors (RIG-1, MDA5 and TLR3), which were found to lead to cell-intrinsic activation of an interferon signature. This in turn produced a protumorigenic microenvironment and was associated with poor patient outcome [63]. Rigorous cell culture experiments combined with RNAseq analysis demonstrated the ability of primary PDAC tumor cell culture supernatants from the DNA methylation-low, RE-activated, IFN sign ‘high’ phenotype pancreatic tumor cells (MC2 type) to reprogram normal pancreatic stromal fibroblasts to a more proinflammatory and tumor-promoting phenotype.

Because of the emerging importance of molecular classifications in PDAC [64,65], investigators performed cell-of-origin and molecular subtyping investigations. These analyses indicated MC2 PDAC tumors to be likely of ductal origin and to at least partially overlap with previously the described squamous-/basal-/quasi-mesenchymal-like subtype signature. By contrast, MC1 methylation ‘high’, IFN ‘sign low’ PDAC tumors more closely resembled acinar cells and partly overlapped with the progenitor-like/classical subtype signature. Of note, normal human pancreatic ductal cells showed enrichment for interferon and dsRNA binding transcriptomic signatures compared to acinar cells, suggestive of a role for dsRNA sensing in shaping normal pancreatic development. Taken together, these findings are consistent with previous data showing a key role for stromal phenotypes in PDAC and show that these phenomena are mechanistically linked via RE-derived dsRNA sensing by innate immune receptors, such as those of the RIG-1-like family. Therapeutic targeting of these pathways may offer opportunities for improved treatment of very aggressive PDAC subtypes.

#### 4.1.2. Colon Cancer

Altered RNA expression of repetitive sequences and retrotransposition has been seen in colorectal cancer, implicating a functional importance of repeat activity in cancer progression [28,37,58,66]. Early studies implicated SATII-derived RNA as substrates for RT activity which results in the formation of dsDNA-RNA hybrids that subsequently reintegrate the genome to induce pericentromeric satellite expansions and genome instability [58]. Subsequently, repeatome profiling in colon cancer cell lines and primary colorectal tumors revealed high expression of LINE-1 and HERV-H repeats and their expression patterns were distinct and associated with different histopathological, genetic, and clinical features [66]. Of note, LINE-1 RNA expression was linked with global methylation status, TP53 mutation (at least in cell lines) and tracked with DNA hypomethylating agent-mediated demethylation in colon cancer cells [66]. Quite strikingly, it has since been shown that the nucleoside reverse transcriptase inhibitor, 3TC, can target the activities of repeat elements in colorectal cancer preclinical models with a preferential effect in p53-mutant cell lines [37]. Indeed, 3TC treatment of TP53 mutant colon cancer cell lines induced decreased motility, anchorage-independent cell growth and in vivo tumor growth, compared to TP53 WT colon cancer cell lines. This was linked to derepression of LINE1 and HSATII RNA consequent to loss of p53 binding and presumably p53 RE suppression activities at these RE. Detailed analysis of 3TC effects on colorectal cancer tumorspheres demonstrated accumulation of immunogenic RNA:DNA hybrids linked with induction of interferon and DNA damage response genes consistent with the activities of endogenous RTE-derived RT at DNA damage sites and in cytosolic nucleic acid signaling via dsDNA products. Importantly, epigenetic and DNA-damaging agents were found to induce repeat RNAs and to have enhanced cytotoxicity with 3TC [37]. In a human phase II trial of single-agent 3TC treatment in metastatic colorectal cancer, clinical benefit was demonstrated in 9 of 32 patients [37]. In sum, this study identifies and clinically validates a novel vulnerability targeting the viral mimicry of repeat elements through either blockade of the reverse transcriptase activity of endogenous RTE or by directly targeting RE-derived RNA such as SATII transcripts, in colorectal cancer.

Interestingly, aberrant expression of HSATII repeats have also been shown in cases of human cytomegalovirus (HCMV)-associated colitis and the HCMV proteins IE1 and IE2 proteins cooperate to induce HSATII RNA, which in turn affects several aspects of the HCMV replication cycle, viral titers and infected-cell processes [67]. It will be interesting to investigate the overlap that may exist between HSATII RNA function in viral infection and cancer, at least in colon tissue.

#### 4.1.3. Ovarian Cancer

In a recent repeatome profile performed across ovarian, pancreatic, and colorectal cell lines, distinct clustering independent of tissue of origin captured by coding gene analysis was observed [30]. By more detailed analysis of ovarian cancer cell lines, HSATII satellite repeat expression was uncovered and found by gene set enrichment analysis (hallmark data sets) to be highly associated with epithelial–mesenchymal transition (EMT) and negatively correlated with interferon (IFN) response genes, indicative of a more aggressive phenotype. Importantly, the relationship of HSATII expression with high EMT and low IFN response genes was also found in RNA-seq of primary ovarian cancers and was associated with significantly shorter survival [30]. Quite strikingly, repeat RNAs were also found enriched in tumor-derived extracellular vesicles that were capable of stimulating monocyte-derived macrophages, demonstrating a mechanism of altering the tumor microenvironment with these viral-like sequences. Suppression of HSATII with antisense locked nucleic acids (LNAs) stimulated the IFN response and induced MHC I expression in ovarian cancer cell lines, thus pointing to a possible means of modulating the repeatome to reestablish antitumor cell immune surveillance [30]. Exactly why HSATII RNA expression leads to an immunosuppressive phenotype was not examined, but one possibility is that HSATII RNA might be sequestered in the nucleus in these cases, thereby circumventing induction of viral mimicry responses [68].

#### 4.1.4. Melanoma

In a study designed to describe the transcriptional landscape changes of primary melanomas of early-, intermediate-, and late-stage tumors and benign precursor lesions, the Greenbaum laboratory identified a distinct, previously unrecognized subset representing high-risk melanomas with poor survival characteristics [69]. Remarkably, this subtype showed TP53 family member anomalies (TP53, TP63 and TP73), epigenome deregulation, and repression of endogenous retrotransposons ((LINEs, SINEs, and ERVs) and viral defense signaling. This high-risk subtype and its prognostic significance was clinically validated in different melanoma cohorts, thereby providing a molecular subtyping framework that takes into account the repeatome for risk prediction in melanoma [69].

#### 4.1.5. Triple Negative Breast Cancer (TNBC)

By a chemical screening approach to systematically investigate the epigenetic dependence of TNBC, a specific vulnerability to the inhibition by MS023 of type I PRMTs (protein arginine methyltransferases) has been identified [70]. Molecular investigations of the underlying cell line-specific dependence allowed validation in human-derived TNBC organoid models. Type I PRMT inhibition alters mRNA splicing, which leads to the expression of Alu sequences that can form cytosolic double-stranded RNA (dsRNA). This in turn triggers an antiviral interferon (IFN)-mediated response that drives TNBC cells that are already stressed due to the elevated preexisting IFN response signature over a threshold to induce cell death, thus validating a novel therapeutic concept in TNBC [70].

#### 4.1.6. Acute Myeloid Leukemia (AML)

In a very rigorous study combining a novel bioinformatics approach integrating repeat and non-repeat expression status and clinical data in AML, the Jenuwein group has shown that AML can be subdivided into ‘high’ versus ‘low’ repeat expression (SINE/ALU, LINE, ERV and satellites) subgroups that differ by biological pathways and prognosis, independently of standard prognostic scores (ELN, European Leukemia Net) [22]. The ‘high repeat’ AML subgroup was characterized by repression of cancer pathways (including metastasis and neoplasms) and activation of a death/apoptosis pathway. By contrast, the ‘low-repeat’ expression AML patient subgroup showed changes in immune response-related pathways such that hypersensitive reaction pathways were activated, whereas infection and inflammation pathways were suppressed. Interestingly, these distinct differences of altered gene expression pathways between ‘high-repeat’ and ‘low-repeat’ AML patient subgroups were in addition to upregulated Toll-like receptor signaling, NFκB activation and interferon signaling, which appeared to be similarly stimulated in both ‘high-repeat’ and ‘low-repeat’ AML patient subgroups compared to the CD34+ (blueprint) control cells [22]. Interestingly, a high repeat-to-gene (R/G) expression ratio identifies AML patient subgroups with a favorable prognosis, whereas a low R/G ratio is prevalent in AML patient subgroups with a poor prognosis. This is in keeping with previous work indicating that TE suppression might be a means for immune evasion in high-risk AML and myelodysplastic syndrome [71].

### 4.2. Therapeutic Intervention for Derepression of Repeat Elements in Cancer: A Novel Approach for Immuno-Oncology?

Anticancer treatments, particularly by epigenetic strategies, can derepress repeat element transcription, which—if associated with the so-called viral mimicry response—have either positive or negative impact on treatment with immune modulating agents [53,54,60]. This has been reviewed extensively elsewhere [31], but merits mention here for the opportunity it offers in relation to improving responses to immune checkpoint inhibitors or for mobilization of the immune system in immunologically ‘cold’ tumors, a major source of treatment resistance or failure in both solid and ‘liquid’ cancers. Additional approaches include targeting of endogenous reverse transcriptase activities, as recently shown in colorectal cancer [37] or indeed, somewhat paradoxically (given its role in RE suppression), induction of p53 that triggers the viral mimicry response by allowing p53 to bind and activate a subset of ERVs [72]. These approaches could be tested alongside other strategies that are gaining traction in this fast-moving field [73].

### 4.3. Repeat Element Activation in Oncology/Onco-Hematology: Biomarker Value?

From the examples given above, it is clear that the cancer ‘repeatome’ has a significant impact on cancer cell hallmarks, cancer-associated immune/stromal phenotypes, and treatment response. Leveraging the ‘repeatome’ for functional precision oncology or in risk prediction is still, however, in its infancy and mechanisms appear complex and context-dependent. Indeed, endogenous retrovirus expression may predict immunotherapy response better than conventional immune signatures in one cohort but not in another [27]. On the other hand, global repeat derepression, including the HSATII satellite repeat, correlates with an immunosuppressive phenotype in colorectal and pancreatic tumors, and this was validated in situ [27]. Taken together, this emphasizes the importance of analyzing the full spectrum of repeat transcription to decode their role in tumor immunity [27].

Repeatome analysis does, however, come with challenges. In particular, it is necessary to consider use of total RNA sequencing to fully capture all potentially relevant repeat element transcripts alongside coding genes [27]. Although poly(A) selection efficiently detects coding genes, most noncoding genes, and limited subsets of repeats, it fails to capture overall repeat expression and coexpression. Alternatively, total RNA expression reveals distinct repeat coexpression subgroups and delivers greater dynamic changes, implying they may serve as better biomarkers of clinical outcomes. In this setting, it is important to note that many repeat element transcripts are not polyadenylated (satellite repeat-derived RNAs, for example [27]) and are thus missed by sequencing strategies that are limited to the poly-A fraction of RNA. Much sequencing data from the TCGA consortium is from poly-A selected RNA, thus rendering repeat element annotation of transcripts suboptimal [27] and leading to missed biomarker information.

An additional avenue of high potential for ‘repeatome’ exploration concerns extracellular vesicles, which are now known to transport and deliver critical molecules, including repeat RNAs, to other cells [74]. EVs are easily accessible for most indications in oncology/onco-hematology, and extending their use for repeatome analysis is a logical next step. This is particularly exciting, since (as discussed above) RE-derived RNAs have been found enriched in tumor-derived extracellular vesicles and these were capable of stimulating monocyte-derived macrophages, thereby revealing a mechanism of altering the tumor microenvironment with these viral-like sequences [30]. Cell free-based assays or liquid biopsy approaches aimed at detecting RE-derived RNA in plasma might also be an option.

## 5. Conclusions

**Figure 4:**
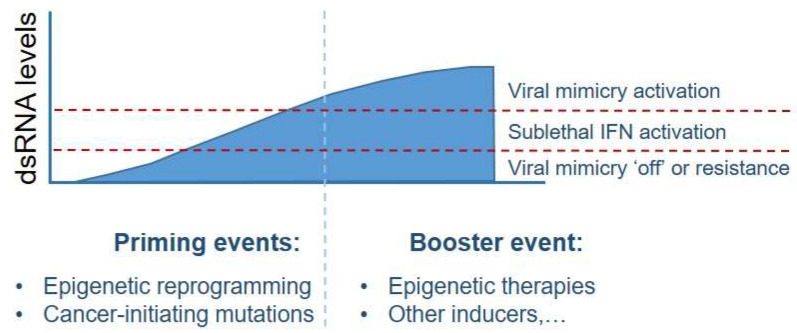
Viral mimicry induces immunogenic cell death in a dsRNA dose-dependent manner. The tolerance threshold for induction of lethal IFN responses in response to dsRNA differs in a cell context-dependent manner. Cancer-initiating mutations or epigenetic malfunctions or drug-induced epigenetic reprogramming act as ‘priming events’ that activate repeat elements and elevation cytosolic dsRNA levels. This increases cancer cell sensitivity to ‘booster events’ that further derepress dsRNAs beyond dsRNA tolerance thresholds. In this setting, cancers with elevated baseline dsRNA levels can be viewed as ‘primed’ for viral mimicry induction. Certain cancers (such as acute myeloid leukemia) have evolved mechanisms to suppress the viral mimicry response and can be referred to as ‘resistant’. Understanding such resistance mechanisms is required to derive new therapeutic strategies for engagement of the viral mimicry response (figure adapted from the review by Chen and colleagues [3]).

In conclusion, RE element transcripts are induced in various physiological and pathological conditions such as inflammation and cancer. A remarkable recent finding is that RE transcripts/dsDNA (generated by endogenous RTE-derived RT activity) can act as cytoplasmic immunostimulatory ligands for activation of innate immune receptor signaling, particularly via the RIG-1 like family, cGAS-STING, and possibly other nucleic acid sensing receptors as well. This in turn leads to nuclear translocation of interferon regulatory factor 3 (IRF3) and IRF7, which together with the transcription factor nuclear factor-κB (NF-κB) induce the expression of type I interferons and other genes. This sterile inflammatory signaling response is emerging as a key means to maintain tissue homeostasis during normal developmental and stress-induced hematopoiesis. Importantly, this pathway appears to be chronically engaged consequent to aberrant ‘awakening’ of RE in cancer, a process that ultimately drives cancer cell-intrinsic phenotypes (EMT, for example) by as yet poorly understood mechanisms of RNA-guided chromatin reprogramming, and immune/stromal microenvironment remodeling through inflammatory responses. Indeed, in certain cancers, RE-derived RNA detection and defense systems appear to be hijacked for protumoral functions, yet in other instances, oncogene signaling appears to drive deep silencing of RE (particularly, retrotransposons) in order to suppress the viral mimicry response in order to evade immune responses. Pharmacological intervention to de-silence or tip the viral mimicry response beyond the sublethal, chronic inflammatory signaling characteristically seen in cancer cells is emerging and offers significant opportunities for treatment innovation (Figure 4).

## Data Availability

Not applicable.
